# Absolute retinal blood flowmeter using a laser Doppler velocimeter combined with adaptive optics

**DOI:** 10.1117/1.JBO.25.11.115002

**Published:** 2020-11-24

**Authors:** Frederic Truffer, Martial Geiser, Marc-Antoine Chappelet, Helene Strese, Gilbert Maître, Serge Amoos, Florent Aptel, Christophe Chiquet

**Affiliations:** a University of Applied Sciences and Arts of Western Switzerland, Institute of System Engineering, Sion, Switzerland; b University Hospital, Department of Ophthalmology, Grenoble, France; c Grenoble Alpes University, Faculty of Humanities, Health, Sport and Society, Grenoble, France; d Inserm, CHU Grenoble Alpes, HP2, Grenoble, France

**Keywords:** laser Doppler velocimetry, retinal blood flow, absolute retinal blood flow, fundus camera

## Abstract

**Significance:** The development of a technique allowing for non-invasive measurement of retinal blood flow (RBF) in humans is needed to understand many retinal vascular diseases (pathophysiology) and evaluate treatment with potential improvement of blood flow.

**Aim:** We developed and validated an absolute laser Doppler velocimeter (LDV) based on an adaptive optical fundus camera that provides simultaneously high-definition images of the fundus vessels and absolute maximal red blood cells (RBCs) velocity to calculate the absolute RBF.

**Approach:** This new absolute LDV is combined with the adaptive optics (AO) fundus camera (rtx1, Imagine Eyes^©^, Orsay, France) outside its optical wavefront correction path. A 4-s recording includes 40 images, each synchronized with two Doppler shift power spectra. Image analysis provides a vessel diameter close to the probing beam, and the velocity of the RBCs in the vessels are extracted from the Doppler spectral analysis. A combination of these values gives an average of the absolute RBF.

**Results:** An *in vitro* experiment consisting of latex microspheres flowing in water through a glass capillary to simulate a blood vessel and *in vivo* measurements on six healthy humans was done to assess the device. In the *in vitro* experiment, the calculated flow varied between 1.75 and 25.9  μL/min and was highly correlated ($r^{2}=0.995$) with the flow imposed by a syringe pump. In the *in vivo* experiment, the error between the flow in the parent vessel and the sum of the flow in the daughter vessels was between −11% and 36% (mean±sd, 5.7±18.5%). RBF in the main temporal retinal veins of healthy subjects varied between 0.9 and 13.2  μL/min.

**Conclusions:** The AO LDV prototype allows for the real-time measurement of absolute RBF derived from the retinal vessel diameter and the maximum RBCs velocity in that vessel.

## 1 Introduction

The inner retina is supplied by the retinal circulation, which is characterized by lower flow than the choroid, high-oxygen extraction, absence of any anastomosis, and autonomic innervation. The retinal blood flow (RBF) adaptation to the perfusion pressure is modulated by a fine autoregulation based on a balance between myogenic (via endothelium) and metabolic (via neurons and glia) mechanisms.^1^ Abnormal variations of RBF have been reported in a variety of ocular diseases including age-related macular degeneration^2^^–^^4^ and glaucoma,^5^ but also in systemic diseases such as diabetes^6^^,^^7^ and systemic hypertension.^8^^–^^10^ Comprehensive knowledge of RBF, essential for understanding the pathophysiology of ocular and systemic diseases, is also the key to the evaluation of therapeutic strategies.^11^

RBF depends on the vascular cross section and the velocity of red blood cells (RBC). Several techniques have been developed to estimate or to measure RBF in the human eye such as fluorescein angiography,^12^ laser Doppler velocimeter (LDV),^13^^–^^17^ Doppler optical coherence tomography,^18^^–^^20^ optical coherence angiography^21^^,^^22^ (OCT-A), and laser Doppler holography^23^ (LDH). Although OCT-A is able to show where blood is circulating, it does not estimate velocity of RBC and thus gives no information about the flow rate.^24^ LDH has the potential to evaluate the velocity within the vessel but mainly depends on the frame rate, which must be in the order of 100 kHz. Although the canon laser blood flowmeter^17^ systems allow for quantification of blood flow using one acquisition, the LDV systems developed so far allow for the independent acquisition of blood velocity and retinal vascular diameters.^14^^–^^16^^,^^25^^,^^26^ Simultaneous measurements of retinal vascular diameters and velocity are highly advisable due to physiological variability of these parameters.

The measurement of blood vessel diameter is based on fundus images combined with image analysis using densitometry.^27^^,^^28^ The recent and innovative technology using adaptive optics (AO) aims at correcting low-order and high-order ocular aberrations, enhances performance of the optical systems, and allows for high-resolution imaging of retinal vessels. For instance, the rtx1 instrument provides *in vivo* retinal images with high lateral resolution (1.6  μm per pixel) and a quantitative analysis of microvascular structures, especially the measurement of retinal arteriolar wall thickness.^29^

To overcome the limitations of the present technologies, we developed an approach based on simultaneous measurements of retinal vessel diameters using AO technology that provides 16-bit images^29^ and is able to measure the inner retinal vessel and blood velocity using bidirectional LDV. In this paper, we report *in vitro* and *in vivo* experiments investigating the validity of blood flow rate measured by this new prototype (aoLDV).

## 2 Materials and Methods

### 2.1 Description of the Instrument

#### 2.1.1 Camera with adaptive optics

Images were obtained using the commercially available AO retinal camera,^30^^,^^31^ which measures and corrects wavefront aberrations with a 750-nm super luminescent diode source and an AO system operating in a closed loop. A 4°×4°  fundus area (i.e., $∼1.2\times 1.2\;{\mathrm{mm}}^{2}$ in emmetropic eyes) is illuminated at 840 nm by a light emitting diode with low temporal coherence, and a stack of 40 fundus images is acquired in 4 s (10 images per second) by a charge-coupled device camera. The rtx1 provides a continuous fundus image that is used to align the probing beam. Its intensity is 100  μW at the cornea, well below the maximum permissible exposure of 700  μW at that wavelength (ANSI Z136.1-2000).

#### 2.1.2 Principle of bidirectional laser Doppler velocimetry

**Fig. 1:**
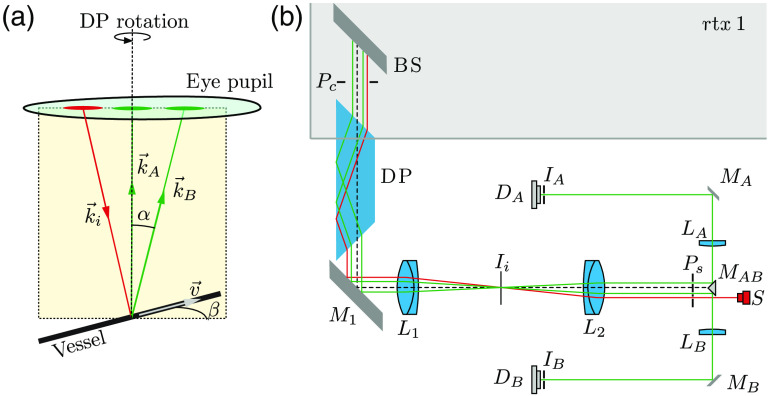
(a) Principle of absolute LDV: ${\vec{k}}_{i}$ represents the probing beam direction and ${\vec{k}}_{A}$ and ${\overset{\to }{k}}_{B}$ are the scattered directions that are selected within the pupil. The three vectors ${\vec{k}}_{i}$, ${\vec{k}}_{A}$, and ${\vec{k}}_{B}$ are on the same plane that makes an angle β with $\vec{v}$. (b) Optical system: except the BS (830 nm RazorEdge Dichroic laser-flat BS, Semrock, USA), which is part of the rtx1, all optical elements are mounted on a separate system that is attached to the rtx1 and aligned with respect to it. $P_{x}$ are the pupil planes, $I_{x}$ are image planes, $M_{x}$ are mirrors, $L_{x}$ are lenses, and $D_{x}$ are detectors. The DOE (on a slider not seen in the image) is placed at $P_{s}$.

**Fig. 2:**
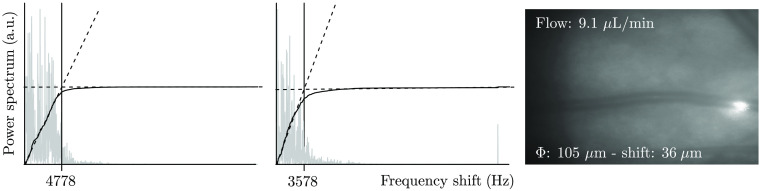
Single-measurement example. Graphs: graphical explanation of the procedure used to find the maximum frequency shift of both signals. The function $r_{c}[i(f)]$ (solid black line) is derived from the power spectrum (gray line). Dashed lines are fitted lines of $r_{c}[i(f)]$ at the beginning and at the end of the frequency domain. The crossing of both fitted lines defines the maximum frequency shift. Image: corresponding fundus image with the calculated value of the flow and the diameter.

The principle of absolute LDV for the human eye was first published by Riva et al.^14^ One probing beam with direction ${\vec{k}}_{i}$ is focused on a blood vessel, in which laminar flow is assumed [Fig. 1(a)]. Light is backscattered and Doppler frequency shifted due to the movement of RBCs within the vessel. Two scattered beams, A and B, are selected with a dedicated pupil so that the angle α between both beams is defined by the optical system and the length of the eye. The vector of the principal ray of each beam and the velocity of the blood in the vessel are all in the same plane. Assuming a quadratic velocity profile^32^ of the RBCs moving in the vessel, the power spectrum of the electronic signal has a theoretical step shape with a sharp edge at the maximum frequency shift (see spectra of Fig. 2). The maximum frequency shift corresponds to the maximum velocity of the blood $v_{\max}$ in the center of the vessel. In this case, it can be shown that

$$v_{\max}=\lambda \cdot \frac{\left|f_{A}-f_{B}\right|}{n\cdot \alpha \cdot \cos\;\beta },$$ (1)

where $f_{A}$ and $f_{B}$ are the maximum frequency shifts of beams A and B, respectively, λ is the wavelength of the laser source, and n is the refractive index of the plasma surrounding the RBCs. The angle α, between the two scattered beams, is calculated using the formula α=arctan(x/L), where x represents the distance between the beam position (A and B, Fig. 1) at the eye pupil and L is the axial eye length assumed to be 23.95 mm^33^ and is set identical for all subjects. By observing the line of the probing beam on the fundus image acquired by the rtx1 camera, the angle β [Fig. 1(a)] is adjusted to zero by rotating a Dove prism (DP) [Fig. 1(b)] so that it has no influence on the calculation of $v_{\max}$. In practice, an angle β up to 10 deg (inducing an error of 1.5%) is acceptable to obtain a reliable measurement of $v_{\max}$.

#### 2.1.3 Optical layout of the velocimeter

The LDV optical system is introduced inside the illumination path with the beam splitter (BS) [Fig. 1(b)] and is located outside the adaptive optical path of the rtx1. A 830-nm laser source S focused at infinity enters the optical system close to the edge of the pupil of the instrument, passes through the LDV and rtx1 optical systems to enter the pupil of the subject, and focuses on a vessel. The probing beam diameter on the retina is about 50  μm. Part of this light is backscattered by the vessel and the RBCs and goes back through the pupil of the eye to the same optical system up to the image of the pupil plane $P_{s}$ where two holes select the two specific directions ${\vec{k}}_{A}$ and ${\vec{k}}_{B}$. A small reflecting prism $M_{AB}$ deflects each beam to lenses $L_{A}$ and $L_{B}$, which focus each beam on a pinhole at the image plane $I_{A}$ and $I_{B}$ corresponding to about 100  μm at the retinal plane. Detectors $D_{A}$ and $D_{B}$ (APD C5460, Hamamatsu) are placed just behind the pinholes. A diffractive element (DOE) placed at $P_{s}$ converts the spot in the fundus into a line. Then the (DP) is rotated to visually align this line with the vessel on the screen of the rtx1 (minimizing β). Lenses $L_{1}$ and $L_{2}$ form a telescope that relays the rtx1 pupil $P_{c}$ to the pupils $P_{s}$ of the LDV optical system with a unity magnification.

#### 2.1.4 Signal acquisition and electronics

The electrical signals from the two detectors are connected to an acquisition card (USB-6356, National Instruments) and are sampled at 120 kHz and Fourier analyzed. The direct current corresponding to the light intensity of a collected scattered light beam was removed by an analog filtering before the acquisition. During each of the 40 acquisitions of 100 ms, 45 ms are used to acquire one high-resolution image (rtx1), 30 ms for one pair of Doppler spectra (LDV) and the rest for the closed-loop AO. The trigger button of the rtx1 simultaneously starts recording the images on the rtx1 and the Doppler acquisition, which is controlled by a dedicated LabVIEW software based on a version developed for a laser Doppler flowmeter.^34^

#### 2.1.5 Signal analysis

The power spectrum is given by $\left\{s_{k}\right\}$, where 1≤k≤N. Considering the ideal case of a rectangular distribution of the power spectrum, the normalized function $r_{c}$ of partial summation:

$$r_{c}(i)=\frac{\overset{k=i}{\underset{k=1}{\sum }}s_{k}}{\max{\left(r_{c}\right)}^{2}}$$ (2)

will linearly increase up to the maximum power spectrum and then remain constant (Fig. 2 dashed lines). First, the horizontal line is found with a linear fit of the last 1/3 of the $\left\{s_{k}\right\}$, for which only noise is expected. Then a second linear fit is done from k=1 to $k_{x}$, corresponding to a 10% change between the first linear fit and the $r_{c}$ function. The intersection of both fitted lines is assumed to be the maximum frequency shift of the spectrum.

#### 2.1.6 Image analysis

**Fig. 3:**
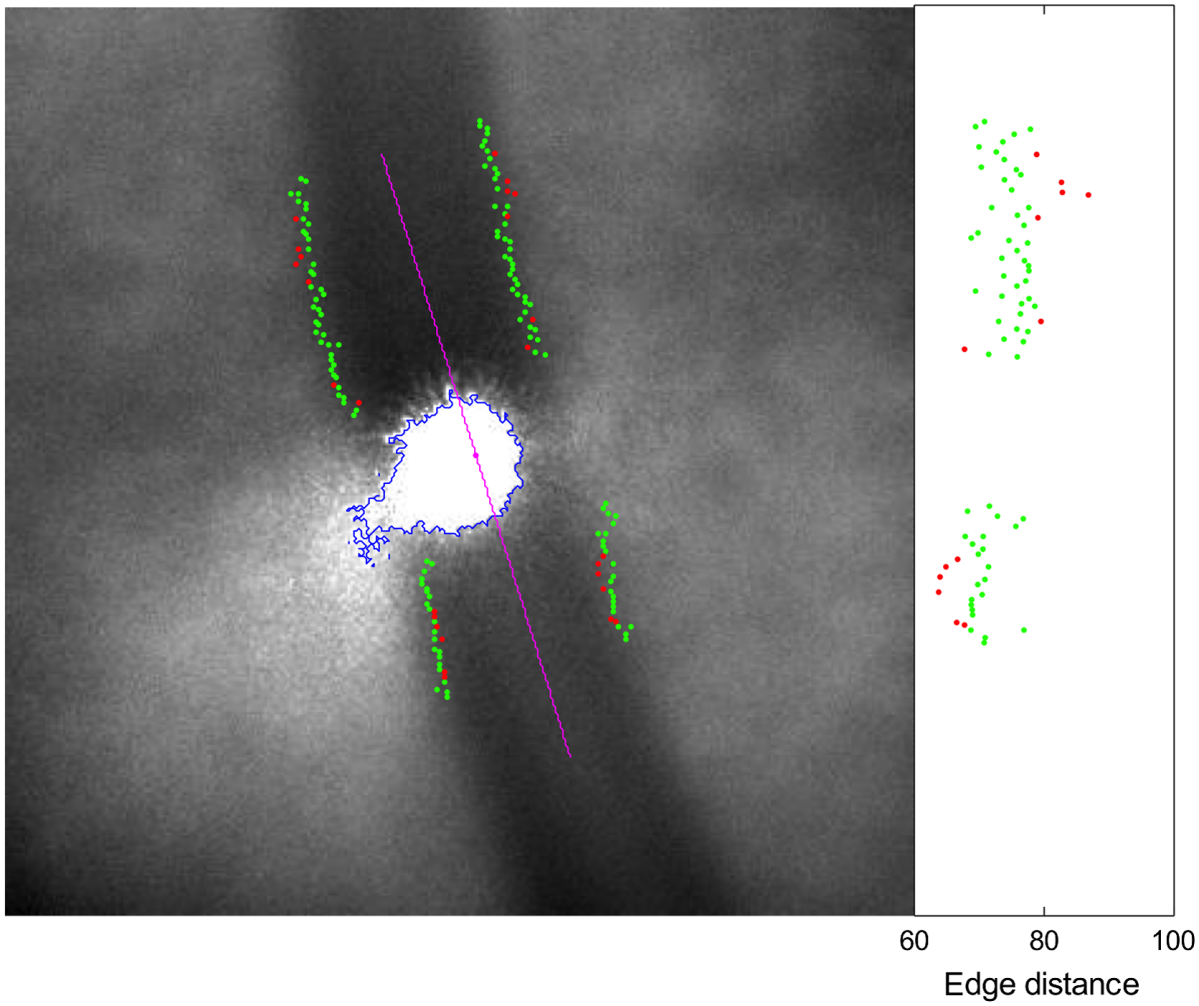
Results of the image processing steps: the detected probing beam spot is the region bordered by the blue points. The spot centroid is in the center of a 360×360  pixels region of interest. The detected vessel orientation and position is represented by the straight line in magenta and the edges by the green and red points superimposed on the image. The distance between each pair of points is drawn in the graph on the right. Points drawn in red correspond to aberrant edge distances (values outside mean±3  sd). The mean distance between the green points is used to estimate the vessel diameter; 73.3 pixels in that case.

The measurement of the vessel diameter close to the probing spot was implemented in MATLAB. Images are 16-bit gray scale with a resolution of 1392×1040  pixels, corresponding to roughly 1.6  μm per pixel at the fundus. First, a threshold is applied at 80% of the maximal image intensity, and the probing beam spot is detected as the largest connected component. Then a 360×360  pixels square region of interest is set around the spot centroid, and a second-order polynomial is fitted to the intensity data to model the low-frequency variations due, among others, to the non-uniform illumination. The subsequent processing is applied on the difference between the original intensity values and the fitted model. The vessel orientation and the offset of its centerline with respect to the spot centroid are found by correlation with 24 precomputed synthetic vessel templates (15 deg increment). Finally, with this rough vessel orientation, image profiles are extracted across the vessel centerline with an interprofile distance of two pixels. The distance in pixels between the vessel edges is determined for each profile. As last step, the vessel diameter is estimated as the mean value of the non-aberrant edge distances determined on each profile (see Fig. 3). The conversion from n pixels to physical distance d is based on the publication of Bennet^35^ and was provided by Imagine Eyes^©^:

$$d=n\frac{19.269(L-1.82)}{373.87}\;\mu m,$$ (3)

where L is the axial length in millimeters of the eye under examination.

#### 2.1.7 Calculation of mean flow rate

Assuming a Poiseuille flow in a cylindrical cross section, the mean flow rate was calculated using the following equation:

$$F=\pi {\left(\frac{d}{2}\right)}^{2}\cdot \frac{1}{2}v_{\max},$$ (4)

where d is the diameter of the vessel obtained from the image analysis and $v_{\max}$ is the maximum velocity of the RBCs in the vessel, which is derived from Eq. (1). The blood flow rate F deviates by about 4% per 1 mm change in eye length.

#### 2.1.8 Measurement procedure

The subject was seated in front of the rtx1 and looked inside the rtx1 objective. A high-resolution image of the retina was displayed by the rtx1 to observe the selected vessel and the LDV probing beam. To observe retinal vessels close to the optic nerve head or outside the posterior pole, the range of movement of the internal rtx1 fixation point was not large enough, and therefore, the left eye was fixating on an external point, positioned 1.5 m behind the rtx1.

Once the probing beam was moved on a first- or second-order retinal vessel in the temporal part of the fundus, the DOE was pushed into the optical path, converting the point into a line, which was then aligned with the retinal vessel by rotating the DP. Finally, the DOE was pulled out.

The signals from the detectors were also connected to a loud speaker, so cardiac pulsed sound could be heard if the beam was focused on a retinal artery. The longitudinal beam position on the retinal vessel was optimized by varying the imaging focus plane until the spectra showed a clear cutoff (see Sec. 4) and the acoustic signal corresponded to a Doppler sound. The lateral position of the beam was adjusted with the external fixation point within about 20  μm.

#### 2.1.9 In Vitro measurements on latex microspheres flowing in a glass capillary

We connected a glass capillary (drummond microcaps, inner diameter 200  μm) via a plastic tube to a syringe pump (Agilia SP, Fresenius Kabi, USA). Latex microspheres (1  μm in diameter, Polysciences Europe GmbH, Eppelheim, Germany) suspended in water (180  μL of microsphere in 35 mL of water) were pushed through the capillary with the syringe. Further, a focusing lens (F=25.4  mm, LB1761-B, Thorlabs) was mounted to focus the collimated probing beam on the capillary. This capillary-lens assembly was placed in front of the rtx1 objective. The vessel-simulating capillary was displayed on the rtx1 screen.

Linearity was asssed by comparing the Doppler measurement of the maximum velocity of microspheres with the precise flow set by the syringe pump.

#### 2.1.10 In Vivo measurements on venous junctions

The study was conducted in accordance with the declaration of Helsinki for research involving human subjects and adhered to Good Clinical Practice guidelines. Written informed consent was obtained from the subjects after explaning the study. The study protocol was approved by the local Institutional Review Board (IRB #6705). For this exploratory study, no optimal sample was calculated, but six healthy subjects with a very good fixation ability, between the age of 27 and 58, participated in this study. A complete ocular examination, including slit lamp biomicroscopy, indirect funduscopy, fundus photography, and axial length measurement partial coherence interferometry (Zeiss IOL Master, Carl Zeiss Meditec Inc, Dublin, CA, USA), was conducted before the beginning of the study. Velocity measurements were conducted after dilatation of the right eye using a 1% topical tropicamide (Théa, Clermont-Ferrand, France). The rtx1 allows for an easy superposition of the pupil of the eye with that of the instrument.

**Fig. 4:**
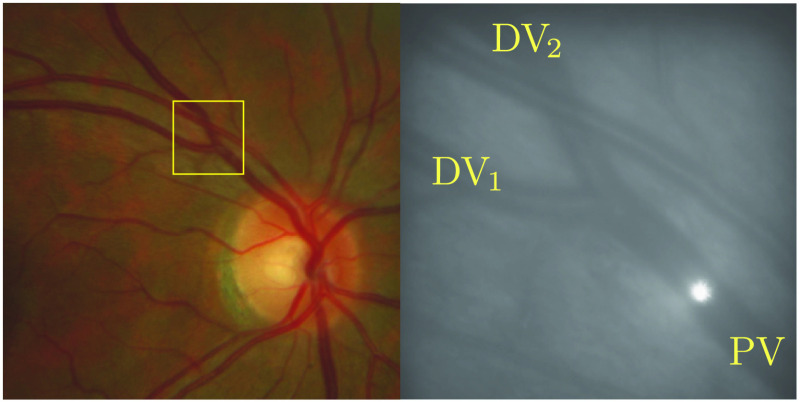
Fundus image and corresponding AO raw image obtained with the rtx1 of a bifurcation measured with aoLDV. PV is the principal vein that furcates into daugther veins ${\mathrm{DV}}_{1}$ and ${\mathrm{DV}}_{2}$.

To test the accuracy of the LDV instrument, blood flow in a retinal venous junction of each human volunteers was measured before the junction and in the two daughter vessels. The measurement before the junction was compared with the sum of the measurements of the daughter vessels to assert their equality. An exemple of a vein and its daughter veins is illustrated in Fig. 4.

## 3 Results

### 3.1 In Vitro Measurements on Latex Microspheres Flowing in a Glass Capillary

**Fig. 5:**
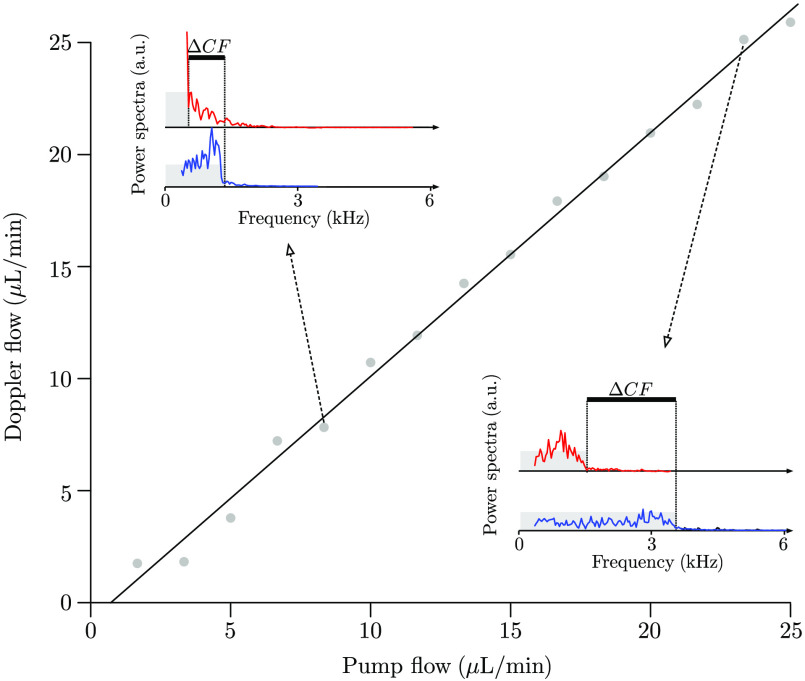
Comparison between the difference in frequency shifts (ΔCFs) obtained with the aoLDV on microspheres flowing in the capillary tube and the flow given by the syringe-pump (correlation coefficient $r^{2}=0.995$). On the power spectra, the peak before the cutoff is due to the light intensity being stronger in the center of the capillary than at the edges. Note that the total power spectrum (area under the curve) remained constant with the increase in velocity.

The Doppler measurements performed on the glass capillary were found to be between 0.92 and 13.3  mm/s for the velocity and between 1.75 and 25.9  μL/min for the flow rate. The measurements were highly correlated (slope equals 1.09, $r^{2}=0.995$, p<0.0001, see Fig. 5) with the flow imposed by the syringe pump.

### 3.2 In Vivo Measurements on Venous Junctions

Measured diameters of the retinal veins varied between 89 and 129  μm (mean 104  μm) (Table 1). The velocity ranged from 2.3 to 21.1  mm/s (mean 11.0  mm/s). The blood flow values in the daughter and parent vessels ranged from 0.9 to 13.2  μL/min (mean 5.7  μL/min). The standard deviation of $\Delta F/F_{\mathrm{PV}}$ was 18.5%.

**Table 1:** Measurements of RBF at retinal venous bifurcation in healthy subjects. ΔF is the difference between the flow in the parent vessel ($F_{\mathrm{PV}}$) and the sum of the flow in the daughter vessels ($F_{\mathrm{DV}1}$ and $F_{\mathrm{DV}2}$): $\Delta F=F_{\mathrm{PV}}-(F_{\mathrm{DV}1}+F_{\mathrm{DV}2})$. Analysis was done automatically. Flow units are μL/min.

Subject	A	B	C	D	E	F
$F_{\mathrm{DV}1}$	1.4	0.9	8.6	4	10.4	8.3
$F_{\mathrm{DV}2}$	3.4	1	6.8	3	1.7	3.5
$F_{\mathrm{PV}}$	4.6	2.1	11.3	5.9	12.8	13.2
$\Delta F/F_{\mathrm{PV}}$ (%)	4.8	−8.3	36.1	19	−6.3	−10.7

## 4 Discussion

The development of this new prototype combining LDV and high-resolution AO imaging showed that (1) the LDV could be added using a complementary optical pathway; (2) optoelectronic development allowed for synchronization between both devices and therefore allowed for AO images and Doppler spectrum acquisitions every 0.1 s; (3) experimental measurements using glass capillary and microspheres showed high correlations between measurements and imposed values; and (4) preliminary measurements in human retinal vessels showed results with acceptable errors.

### 4.1 In Vitro Measurements on Latex Microspheres Flowing in a Glass Capillary

To mimic a 130-μm human retinal vessel, a 200-μm glass capillary with a lens of 25.4 mm focal length was used. Capillaries in air with larger diameters would be preferable because they disturb the AO of the rtx1 less than smaller diameters. Our results show a clear linear relationship between the flow calculated with the Doppler measurements and the flow rate given by the syringe pump.

### 4.2 In Vivo Measurements on Venous Junctions

Our measurements of RBF were in the same range as those reported in previous studies of retinal vessels with similar diameters. Using a device based on the same fundamental principle, a bidirectional laser Doppler velocimeter, and monochromatic fundus photographs, Riva et al.^16^ measured retinal vessel diameters between 64 and 177  μm, corresponding to flow rate between 1 and 20  μL/min, which is consistent with our measurements. Yoshida et al.^36^ used a canon laser blood flowmeter (CLBF100) and measured the blood flow rate to be between 3.2 and 14.4  μL/min (mean 8.3  μL/min) in retinal veins with a diameter ranging from 98 to 166  μm (mean, 138  μm) (see Table 2). In their study, Werkmeister et al.^18^ measured retinal veins between 84 and 172  μm and found RBF between 1.9 and 18.7  μL/min with dual-beam bidirectional Doppler Fourier-domain optical coherence tomography.

**Table 2:** Comparaison between our measurements and the results from different publications.

Publ.	Techn.	Subj. Nb	Age (year)	Eyes Nb	Veins Nb	Dia (μm)	Vel (mm/s)	Flow (μL/min)
	aoLDV	6	29 (25–31)	6	18	(89–129)	0.9–13.3	(121)
16	LDV	7	34 (20–45)	12	66	(64–177)	(0.5–3.6)	(120)
37	LDV	1	(21–43)	—	—	156±20	1.71–0.33	12.5±3.5
38	LDV	12	27±5	—	—	(132–176)	(1.2–2.1)	(7.818.7)
15	LDV	64	31.8±6.5	—	—	(90–185)	(0.8–2.4)	12.2±7^a^
								17.9±7.1^b^
36	LBF	6	(28–43)	12	18	(98–166)	—	(3–14)
18	FD-OCT	10	29 (19–35)	10	30	(84–172)	—	(2–19)
20	FD-OCT	4	(20–30)	—	73	(60–160)	—	(1–16)

Mean (min – max) or mean ± standard deviation.

a Temporal sup.

b Temporal inf.

### 4.3 aoLDV Advantages and Feasibility

aoLDV offers the possibility to perform very precise measurements of the inner diameter of vessels. Averaging 40 raw images, obtained from one measurement with the rtx1, each with a lateral pixel-resolution of 1.6  μm,^39^ gives a better lateral pixel-resolution of 0.8  μm.^40^

In this study, raw images were used to calculate the diameter since each image corresponds to a pair of Doppler spectra. In our study, 94 out of 480 raw images (19.6%) were excluded from analysis, 31 (6.5%) because they could not be analyzed (mainly due to blinks) and 63 (13.1%) because the probing beam was not on the vessel. At the present time, no method to quantitatively assess perfusion is used in routine practice. This is due to the difficulties in performing RBF *in vivo*.

The evaluation of a new method of RBF measurement is difficult in the absence of a gold standard. An LDV system was implemented by canon (CLBF100),^36^ but only a few instruments were sold. Nevertheless, LDV has not reached routine clinical practice, in part because of alignement difficulties and because of the relative imprecision of diameter measurements.^41^ The spatial resolution is limited by the quality of the eye under observation.

OCT scanning is becoming standard in clinical optical imaging.^42^ OCT has a lateral resolution of around 12 to 20  μm and an axial resolution between 2.3 and 7  μm. The price of such cameras is about five times higher than classical fundus cameras, but they offer many advantages of which the most interesting is its ability to build axial slices of the fundus. By adapting the detection, OCT allows for Doppler measurement. However, the speed of the scanning limits the range of measurable velocities. Our proposed system encompasses this shortcoming since we do not scan the retina.

### 4.4 aoLDV Limitations

**Fig. 6:**
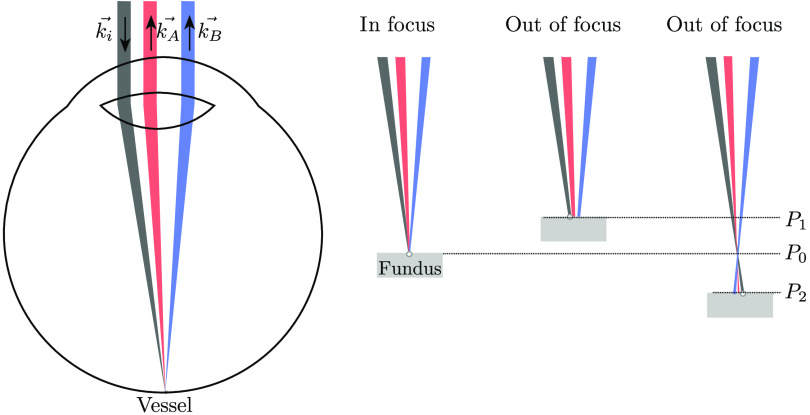
Illustration of the parallax phenomenon, which explains that one or both scattered collected beams (${\vec{k}}_{A}$ and ${\vec{k}}_{B}$) can be out of focus while the laser beam (${\vec{k}}_{i}$) is focused on the vessel (circle on the fovea). $P_{0}$ is the image plane of the rtx1, and $P_{0}$, $P_{1}$, or $P_{2}$ are possible image planes of the LDV optical system.

We have encountered some limitations for the measurements of $v_{\max}$ on human subjects, including the need for a good ocular fixation for 4 s to ensure that the probing and detection beams, respectively, hit and come back from the center of the vessel (Fig. 6). Owing to slight eye movements, we may underestimate $v_{\max}$ by measuring velocities of RBCs close to the vessel wall (lower than $v_{\max}$) or by adding Doppler shifts associated with the surrounding tissue.

The AO live video of the fundus allowed us to verify the exact location of the laser beam on the vessel, but it did not provide the detection plane of the aoLDV, which could be different due to the AO adaptation.

Bidirectional LDV measurement accuracy strongly depends on the plane of focus (conjugate to the detection plane) of the probing beam as well as A and B beams (Fig. 6) and the position of the probing beam, which should be exactly on the vessel center. This causes a parallax problem, which occurs when the image plane of the LDV is not on the same image plane as the rtx1 (Fig. 6). Obtaining a usefull pair of good spectra was a strong technical limitation. The constraint of the existing fundus camera rtx1 prevents us from using the path of the AO for the LDV, leaving this path uncorrected for eye aberrations. To overcome this parallax problem, we manually change the refraction correction of the rtx1 between −0.5 and 3 diopters to find the best correction to match the detection plane. These settings were time-consuming, 1 h for one measurement of a bifurcation.

The power spectrum from a glass capillary with a flowing fluid is typically a step function, in which the maximum of the frequency shift correponds to $v_{\max}$ (cutt-off frequency). The quality of the *in vitro* spectrum obtained with the moving light scattering microspheres was excellent, and the cutoff frequency was always well-defined. Another difficulty was calculating the precise cutoff frequency of the *in vivo* spectrum, which, most of the time, did not have a clear step shape. Thus $v_{\max}$ extraction was dependent on the algorithm to calculate the maximum of the frequency shift.

### 4.5 Clinical and Technical Perspectives

More studies with a larger series of subjects are needed to evaluate the reproducibility and to estimate the total RBF after measuring the main retinal vessels near the optic nerve disc. To allow for examination of both eyes with the rtx1, an external fixation target should be designed. A more robust image analysis with deep learning would reject fewer images. The development of software combining data from the Doppler spectra, the quality of AO images, and the exact location of the probing beam on the retinal vessel is in progress and will allow for real-time evaluation of measurements.

Assuming a correct focus, two illuminating beams $-{\overset{\to }{k}}_{A}$ and $-{\overset{\to }{k}}_{B}$ instead of collected scattered beams must hit at the fovea the same point as the probing beam ${\vec{k}}_{i}$ (Fig 6). This would solve the parallax issue.

## 5 Conclusion

In conclusion, this aoLDV prototype allows for simultaneous measurement of the inner retinal diameter and the velocity of the RBC within that vessel, which allows for real-time calculation of absolute blood flow rate. The main limitations of this prototype were due to optomechanical constraints, which prevents the use of the optical path of AO for the LDV. This produced occasional discrepancies between the focal planes of the AO and LDV optical paths (parallax effect), which suppressed Doppler signal in one or both channels. Improving the analysis method and solving the parallax issue will offer a high potential for examining RBF abnormalities in patients with ocular or systemic diseases. The results obtained in healthy subjects are promising.
